# Apple Juice, Manure and Whey Concentration with Forward Osmosis Using Electrospun Supported Thin-Film Composite Membranes

**DOI:** 10.3390/membranes12050456

**Published:** 2022-04-24

**Authors:** Kitty Nijmeijer, Pelin Oymaci, Sjoukje Lubach, Zandrie Borneman

**Affiliations:** Membrane Materials and Processes, Department of Chemical Engineering and Chemistry, Eindhoven University of Technology, P.O. Box 513, 5600 MB Eindhoven, The Netherlands; p.oymaci@tue.nl (P.O.); s.lubach@tue.nl (S.L.); z.borneman@tue.nl (Z.B.)

**Keywords:** forward osmosis, electrospun thin-film composite (TFC) membrane, whey, apple juice, manure

## Abstract

Forward osmosis (FO), using the osmotic pressure difference over a membrane to remove water, can treat highly foul streams and can reach high concentration factors. In this work, electrospun TFC membranes with a very porous open support (porosity: 82.3%; mean flow pore size: 2.9 µm), a dense PA-separating layer (thickness: 0.63 µm) covalently attached to the support and, at 0.29 g/L, having a very low specific reverse salt flux (4 to 12 times lower than commercial membranes) are developed, and their FO performance for the concentration of apple juice, manure and whey is evaluated. Apple juice is a low-fouling feed. Manure concentration fouls the membrane, but this results in only a small decrease in overall water flux. Whey concentration results in instantaneous, very severe fouling and flux decline (especially at high DS concentrations) due to protein salting-out effects in the boundary layer of the membrane, causing a high drag force resulting in lower water flux. For all streams, concentration factors of approximately two can be obtained, which is realistic for industrial applications.

## 1. Introduction

Concentration of liquid streams is an important process to valorize rest streams in various industries, such as the food, agricultural and dairy industry. Juice, manure and whey are typical examples of streams that usually require concentration for longer shelf life as well as to reduce storage space and packaging, transport or discharging costs [1,2]. Evaporation is a widely used technique to remove excess water from dilute streams; however, it is not only very energy intensive, it can also lead to deterioration of the food products of interest due to the higher temperatures involved. Evaporation is often replaced by membrane processes such as nanofiltration (NF) or reverse osmosis (RO) that do not require high temperatures and are 10 times more energy efficient than evaporation [3]. Due to the highly fouling nature of the feed streams though, the concentration efficiency of such processes is low and maintenance costs are high.

**Figure 1:**
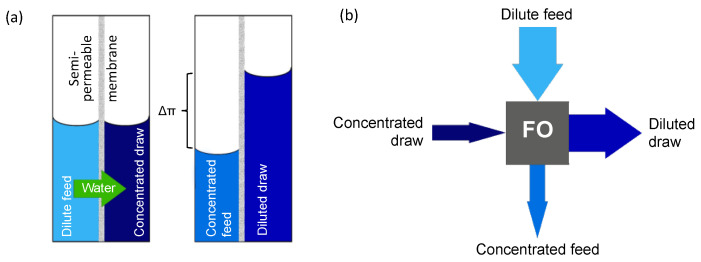
(**a**) Principle of FO using the osmotic pressure difference between two solutions to concentrate a dilute feed solution; (**b**) FO process.

In recent years, forward osmosis (FO) has emerged as an alternative membrane process for concentration. Unlike the pressure-driven membrane processes mentioned above, FO only uses the osmotic pressure difference (chemical potential difference) over the membrane as driving force to draw water from the diluted feed stream (e.g., juice, manure or whey) into a high-osmotic-pressure draw solution (DS) (Figure 1).

Without the need to apply any additional pressure or high crossflow velocities, FO is an energy efficient process that is very suitable for shear sensitive components. It is also less susceptible to fouling, which means less intensive pre-treatment is required, and it allows higher concentration efficiencies [3,4,5]. Obviously, after use, regeneration of the DS to remove additional water and restore its high osmotic pressure is required before reuse [6].

Polyamide (PA) thin-film composite (TFC) membranes are state-of-the-art membranes used in many membrane-based processes (e.g., desalination and wastewater treatment) [7,8]. Due to the self-limiting character of interfacial polymerization (IP), application of a PA top layer on a porous support using IP results in very thin, defect-free layers. The properties of the IP layer can be easily adapted by changing the chemistries of the monomers used in IP. Current commercial TFC membranes are produced at large industrial scale and have superior permeability and selectivity [7,8]. Recent studies investigating the effectiveness of FO to concentrate juices, wastewater and whey solutions mostly used such PA membranes [7,8,9,10,11].

Even though FO is effective in concentrating liquid rest streams, there are still significant challenges. Although less sensitive to fouling than pressure-driven membrane processes, studies show that fouling could have a big impact on performance [1,12]. Anti-fouling membrane coatings (e.g., of poly dopamine) [13], the incorporation of nanoparticles in the membrane [14,15] or the use of zwitterionic membrane moieties [16] can help to control fouling, but commercialization of such products may take long and often involves very intricate synthesis processes and expensive materials [17]. Ibrar et al. [17] performed an extensive review on fouling in FO and concluded that, although data using real wastewater is limited, fouling in FO is mostly reversible, and flux can often be restored using high crossflow velocities or improved hydrodynamics. If irreversible fouling (e.g., biofouling) takes place, chemical cleaning is required. In a recent paper, Oymaci et al. [18] found that the salt concentration gradient over the membrane not only influences the water flux, but it also significantly impacts the type of fouling, and that the presence of a small amount of salt in the feed solution (FS) causes irreversible fouling due to salting in/out effects.

However, the major challenges in FO are to reduce the high reverse salt flux from draw to FS and to diminish internal concentration polarization (ICP) in the porous support of the membrane [6]. High specific reverse salt flux through the selective membrane layer increases the salinity of the final product feed stream, thus deteriorating its quality. Because of its low price and abundance, NaCl is the most-used draw solute to concentrate liquid foods. However, due to the small size and high mobility of the associated ions, reverse salt fluxes are generally high, and efforts focus on the development of alternative draw solutes with inherently lower permeabilities [19,20,21].

**Figure 2:**
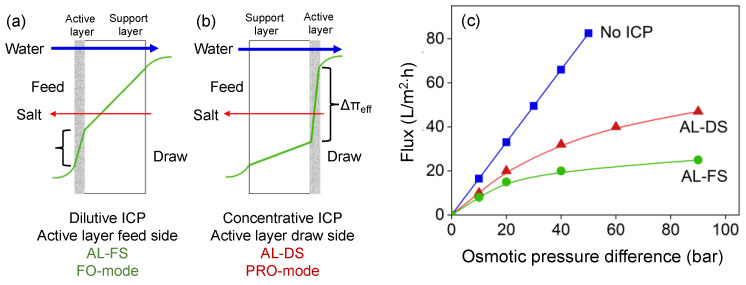
(**a**) Dilutive ICP; (**b**) Concentrative ICP; (**c**) impact of ICP on clean-water flux through the membrane.

ICP occurs in the porous support structure of the membrane; depending on the configuration, a dilutive ICP occurs when the active membrane layer faces the feed side (Figure 2a), or a concentrative ICP occurs when the active membrane layer faces the DS (Figure 2b). In both cases, ICP results in a decrease of the effective osmotic pressure difference over the membrane, thus decreasing water flux through the membrane (Figure 2c). As ICP occurs in the membrane support layer, it cannot be decreased by creating turbulence, and thus requires redesign of a very open and porous membrane support for FO applications [22].

An important improvement to mitigate ICP is the development of new-generation thin-film composite (TFC) membranes with a PA layer on top of a nanofibrous support produced by electrospinning [23,24,25]. The very open structure of such a support decreases ICP significantly, therefore maximizing the effective osmotic pressure difference over the membrane [26]. However, the use of such supports comes with additional challenges: The application of a thin, defect-free PA layer on top of that support is not trivial, as the thin layer must be able to span the large pores without any defects. Moreover, despite advances to reduce ICP, often such newly developed membranes are only investigated measuring clean-water flux data, and real industrial feeds are only used sporadically, meaning the potential of the developed membranes for large-scale industrial FO-processes is not validated.

Thus, the major challenge is the development of a composite membrane with a very open porous support to limit ICP, and a dense, very selective top layer that only allows the passage of water while retaining salt ions to reduce the reverse salt flux using real industrial feed streams.

Following the analysis above, in this research, thin-film composite membranes consisting of an electrospun nanofiber support and an ultrathin PA layer are developed. To enhance the attachment of the coating to the support, the coating and support chemistry is chosen such that the coating is covalently attached to the support. The developed membrane is used in FO to concentrate real industrial apple juice, manure and whey streams. Two NaCl concentrations (1.2 and 3.6 M) with an osmotic pressure of 57 and 193 bar, respectively, are used as DS to research the effect of the ionic strength of the DS. Membrane flux performance is measured, as well as salt retention. Using industrial feeds, the water flux, concentration factors and reverse salt flux values of apple juice, manure and whey solutions of these membranes are discussed as well.

## 2. Experimental Section

### 2.1. Materials

Thin-film composite membranes consisting of an electrospun nanofiber support (NF-TFC) and a thin selective polyamide interfacial polymerization layer were used for all the experiments. For the electrospun support, polysulfone (PSU) was kindly supplied by Solvay (Udel P 3500, Bollate, Italy), and dimethyl acetamide (DMAc) as solvent was supplied by Merck (>99.0%, Darmstadt, Germany). For the polyamide coating on top of the electrospun support, the following chemicals were obtained: m-phenylene diamine (MPD, 99%) and sodium dodecyl sulfate (SDS, >95%) by Sigma Aldrich (Merck, Darmstadt, Germany); camphor sulphonic acid (CSA, 98%) and heptane (extra dry 99% Acroseal) by Across Organics (Thermo Fisher Scientific, Waltham, Massachusetts, United States); triethylamine (TEA, >99%) by Merck (Darmstadt, Germany); trimesoyl chloride (TMC, >98.0%) by TCI chemicals (Tokyo, Japan); and decane (99+%) by Alfa Aesar (Haverhill, Massachusetts, United States). Apple juice (pure, unfiltered) was purchased from Appeltap (Van Appeven, Herten, The Netherlands). Manure effluent water was kindly supplied by Loonbedrijf Kuunders (Deurne, The Netherlands). The manure effluent is the thin fraction of a manure stream that remains after solid–liquid separation and passing through a paper filter, and is normally used as feed in the first RO stage. The whey solution was kindly provided by Duynie Feed (Duynie BV, Alphen aan den Rijn, The Netherlands) as a byproduct of cheese production. Whey solution was initially obtained as sweet whey, but acidified to extend shelf life and prevent rotting, providing sufficient time for experiments. The obtained solution was filtrated before use to remove residuals from production. Sodium chloride (Sanal P^®^) was kindly obtained from Nouryon (Deventer, The Netherlands). Ultrapure water (UPW; 18.2 MΩ·cm) was produced using an ELGA Purelab (VWS, High Wycombe, UK) water purification system and used to prepare all solutions. O-phthalaldehyde (OPA, ≥99.0% for fluorescence), sodium sulfite, formaldehyde solution (37%, ACS reagent), 1 N sodium hydroxide (NaOH) solution (Titripur), anhydrous ammonium sulfate and anhydrous ethylenediaminetetraacetic acid (EDTA, ≥99.0%) were purchased from Sigma-Aldrich (Merck, Darmstadt, Germany). Methanol (MeOH, absolute, AR ≥ 99.8%) was supplied by Biosolve (Valkenswaard, The Netherlands). All chemicals were used as received unless otherwise stated. All solutions were used at ambient pH without any adjustment.

### 2.2. Membrane Preparation

#### 2.2.1. Electrospinning of the Support Membrane

The electrospun PSU nanofiber support was produced with an electrospinner (NS Lab, Elmarco, Liberec, Czech Republic), which is a wire spinning machine equipped with a custom-made air-conditioning unit to set and to keep ambient conditions constant. Prior to use, PSU was dried at 160 °C for 24 h and then dissolved in DMAc that had been dried over molecular sieves for 24 h. The solution was made in a closed bottle that was stirred overnight on a roller bench until a homogeneous viscous liquid was obtained. The PSU solution was then electrospun on a substrate. After spinning, the final electrospun support could be removed from the substrate as a flat sheet. Spinning parameters are presented in Table 1 below.

#### 2.2.2. Polyamide Coating of the Electrospun Support Membrane

**Figure 3:**
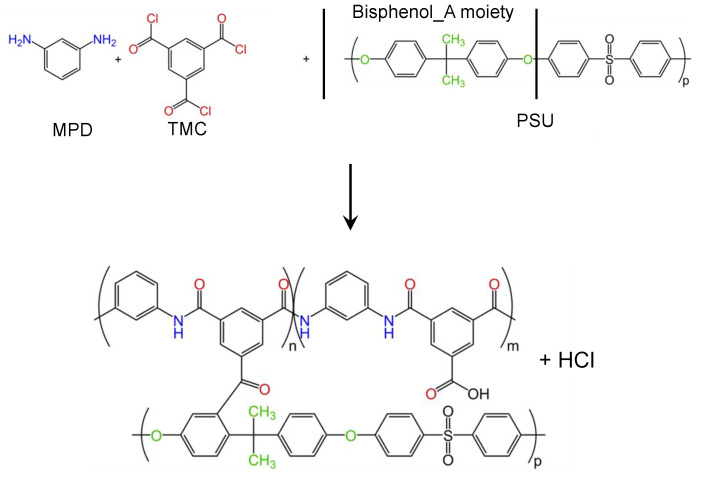
Reaction scheme of the formation of the thin polyamide (MPD + TMC) layer covalently attached to the porous electrospun PSU support.

To apply the PA coating layer while simultaneously allowing large scale production, MPD and TMC are selected as monomers, as these are frequently used in the industrial production of membranes for RO/FO applications [27]. Moreover, in combination with electrospun PSU support, this guarantees covalent attachment of the coating layer to the support material as also described by [28] (Figure 3).

To coat the electrospun support, a framed coating cell was used. The cell had an effective coating area of 44 cm × 32 cm. The electrospun support was positioned on a 1 cm thick aluminum base plate. A rubber seal and a 1 cm thick aluminum frame were subsequently placed on top of the support sample, and then the frame was bolted to the base plate with stainless steel Allen key screws. The coating cell was then soaked in a pure isopropanol bath for 10 min and then transferred into a rinsing bath with ultrapure water. The ultrapure water was replaced two times to rinse out all isopropanol. Then, the sample was soaked for 10 min in a 2 wt.% MPD solution in water, containing also 2 wt.% CSA and 2 wt.% TEA as additives. Next, the coating cell was taken out of the solution and the excess solution was blown off with an air blade at 7 bar of pressurized air. Subsequently, the coating cell with the support sample containing MPD was placed in a leaking tray, and 100 mL of 0.2 wt.% TMC in heptane at −20 °C was poured in the cell on top of the electrospun support. The monomers were allowed to react for 90 s, after which the coating cell was taken out of the solution, the excess heptane was poured off and the sample was heat treated. This heat treatment consisted of placing the sample in several demi water baths in series: first 95 °C for 2 min, then 20 °C for 2 min, again 2 min in 95 °C and a last time for 2 min in 20 °C to cool the sample. After that, the TFC-coated electrospun support was removed from the coating cell, and the membrane was washed three times in demi water to remove all trace chemicals and stored in demi water until use.

### 2.3. Forward Osmosis Performance

**Figure 4:**
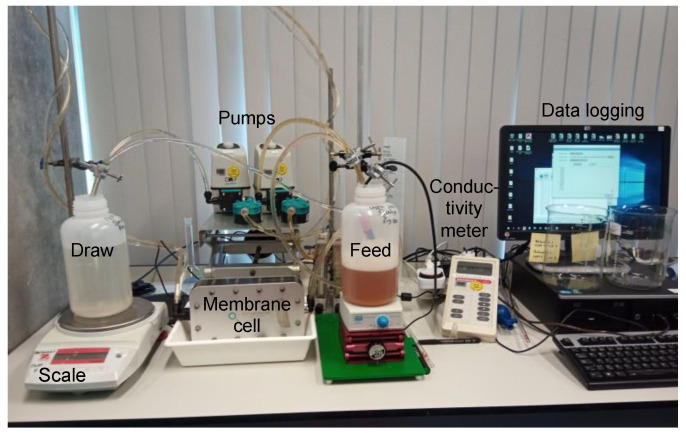
Image of the experimental FO set up.

The FO filtration performance of the TFC-coated electrospun membranes was measured using a crossflow FO filtration system (Figure 4).

A membrane cell (Convergence Industry B.V., Enschede, The Netherlands) with two slits located on both ends of the membrane was used to hold the membrane, with an effective membrane area of 0.006 m^2^ (40 mm width, 150 mm length) and a 5 mm slit height. The membrane was used to filtrate with the active TFC layer facing the FS (FO mode). Diamond-shaped spacers with a thickness of 2 mm were used, two on top of each other at each side of the membrane. UPW, apple juice, manure or whey were used as FS (1.8 L each), which means an initial feed volume/membrane area of 300 L/m^2^. DSs (1 L) were prepared by dissolving NaCl in UPW at an initial concentrations of 1.2 or 3.6 M. The FS and DS were circulated in the system, each at one side of the membrane, by two diaphragm pumps (Liquiport, KNF, Freiburg im Breisgau, Germany). The co-current flowrates were set to 36 ± 2 L/h using a flow meter/regulator (Swagelok, Solon, Ohio, USA), giving a crossflow velocity of 5.7 cm/s (calculated with 85% spacer porosity). Measurements were performed in batch mode at ambient temperature. Five different membranes were first measured with UPW as FS and 1.2 M NaCl as DS to determine their clean-water flux values. Then, apple juice, manure and whey were used as FS, and each stream was concentrated using 1.2 or 3.6 M NaCl as DS. When using 3.6 M NaCl as DS, the solution was refreshed one time during the measurement to keep a high osmotic pressure difference over the membrane and to continue the concentration process. Samples were collected at both the start and the end of the runs for further characterization.

The osmotic pressure difference between DS and FS is based on the calculated osmotic pressures from the DS using the van ’t Hoff Equation (Equation (1)) and the given osmotic pressures from the FS. The osmotic coefficient ϕ is calculated by fitting the osmotic coefficients for 0.3 to 6.0 M NaCl solutions as given by Hamer et al. [29].

(1) $$P_{eff\;\;}=P_{DS\;}-P_{FS}=iCRT\phi -P_{FS,i}\;\cdot CF$$

where *P_eff_* is the effective osmotic pressure (bar), *P_DS_* and *P_FS_* are the osmotic pressures of the *DS* and *FS* (bar), *i* is the van ’t Hoff constant (−), *C* is the molar concentration (mol/L), R is the gas constant (L·bar/K·mol), T the absolute temperature (K), ϕ is the osmotic coefficient (−), *P_FS,i_* is the initial osmotic pressure of the *FS* (bar) and *CF* is the obtained concentration factor (−).

Water flux, J_w_ (L/m^2^·h), was calculated from the collected mass of the permeate volume V_d_ (L) in the DS in a certain time t (h) per membrane area A (0.006 m^2^) (Equation (2)). (The change in conductivity in time was measured at the feed side and used to calculate the reverse salt flux J_s_ (g/m^2^·h) from the change in salt concentration (Equation (3)).

(2) $$J_{w}=\frac{V_{d}}{A\cdot t}$$

(3) $$J_{s}=\frac{c\cdot V_{f}}{A\cdot t}$$

The specific reverse salt flux was calculated as the ratio of the reverse salt-to-water flux J_s_/J_w_ (g/L). The concentration factor was calculated by dividing the initial volume of the FS (1.8 L) by the final volume after concentration.

### 2.4. Sample Characterization

#### 2.4.1. Scanning Electron Microscopy (SEM)

Surface morphologies of the gold or platinum sputter-coated (40 mA, 90 s) samples were characterized by scanning electron microscopy (IT-100, JEOL, Tokyo, Japan), operating at an acceleration voltage of 5 and 15 kV. Samples were dried overnight at room temperature before sputter coating.

#### 2.4.2. Total Organic Carbon (TOC) Measurement

Total organic carbon measurements (TOC-L analyzer, Shimadzu, Kyoto, Japan) were performed to measure the amounts of total carbon (TC) and inorganic carbon (IC) in the feed and DS. All feed and draw samples were diluted 100× and 10×, respectively, prior to analysis.

#### 2.4.3. Atomic Absorption Spectroscopy (AAS)

Atomic absorption spectroscopy (AA-7000, Shimadzu, Kyoto, Japan) with flame detection (detection limit between 0-1 ppm in aqueous solution) was used to determine the amount of potassium in the manure solutions.

#### 2.4.4. Ultraviolet–Visible Spectroscopy (UV–Vis)

Ultraviolet–visible spectroscopy was used to determine the ammonium content of the manure solutions. To determine the concentration, ammonia was complexed with sodium sulfite and OPA in an EDTA buffer, forming a red-rose complex. For this, 3 stock solutions were prepared: (a) 1 g sodium sulfite (Na_2_SO_3_) dissolved in 1 L UPW containing 0.4 mL formaldehyde to prevent the solution from being oxidized. (b) 5.22 g OPA dissolved in 1 L solution with a composition of 500 mL methanol and 500 mL UPW. (c) EDTA–NaOH buffer solution prepared by dissolving 51 g EDTA and 20 g NaOH in 1 L UPW. The EDTA buffer was used to prevent precipitation and to control the reaction [30]. The reaction mixture was prepared by mixing 5 mL stock A with 10 mL stock B followed by diluting until 100 mL with UPW. The complexation reaction was then carried out by mixing 5 mL reaction mixture with 5 mL stock C solution and 5 mL sample in a 15 mL vial. The vial was capped directly and placed in a 55 °C water bath for 10 min. Then the sample was transferred to a UV cuvette and the absorbance at 550 nm was determined and recalculated to the actual ammonia concentration using a calibration curve.

## 3. Results and Discussion

### 3.1. Membrane Preparation

**Figure 5:**
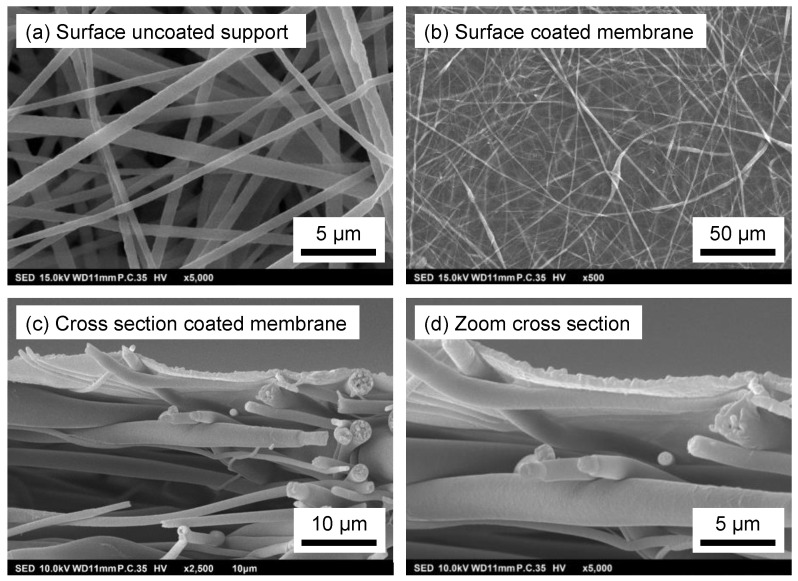
SEM images of the surface of (**a**) the uncoated electrospun PSU support; (**b**) the PA coated PSU support; and (**c**,**d**) the cross-section of the PA-coated PSU support.

The morphology of the developed membranes was investigated by SEM, and the surface images of the uncoated electrospun PSU support and the polyamide coated electrospun PSU support are shown in Figure 5a,b, respectively. The nanofibrous structure of the PSU electrospun support is clearly visible. Fibers are well-separated, forming a nice, highly porous, nonwoven mat without any polymer clusters or droplet formation. The polyamide coating is covalently attached and covers the top surface of the electrospun support, forming a defect-free layer. Figure 5c,d show the cross-section and a zoom of the cross-section, respectively, of the PA-coated electrospun membrane. A very thin PA layer formed through interfacial polymerization spanning the full width of the pores of the support is clearly visible.

The properties of the electrospun support and the ultimate membrane are summarized in Table 2. The support has extremely high porosity, with a mean flow pore size of almost 3 µm, but still a dense, selective PA layer of only 0.63 µm could be applied.

### 3.2. Ideal Membrane Performance Using Clean Water

**Figure 6:**
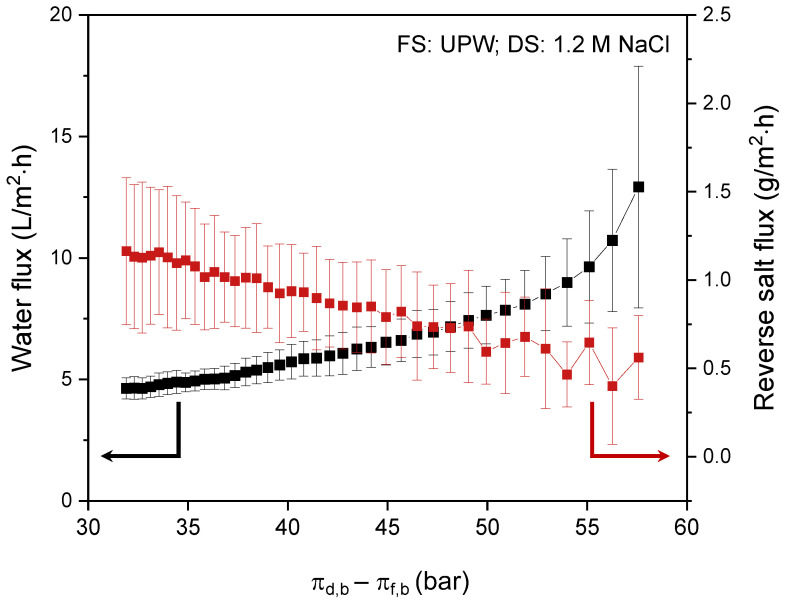
Membrane performance using a 1.2 M NaCl solution DS and UPW as FS: water flux and reverse salt flux as a function of the osmotic pressure difference over the membrane between bulk DS and bulk FS. Average values calculated based on five separate runs.

The ideal performance of the prepared membranes was first investigated using UPW as FS and a 1.2 M NaCl solution as DS. The measured water and reverse salt flux as a function of the actual osmotic pressure difference over the membrane (between bulk FS and bulk DS) is presented in Figure 6.

**Figure 7:**
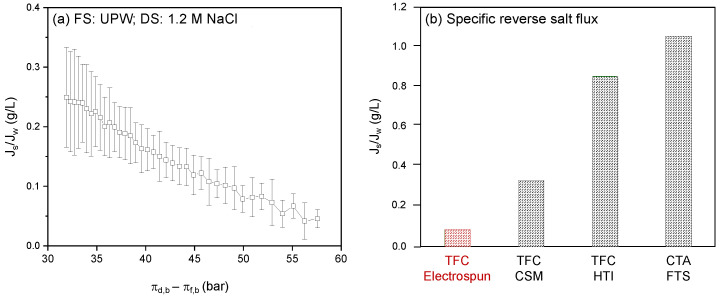
Membrane performance using a 1.2 M NaCl solution DS and UPW as FS. (**a**) Specific reverse salt flux as a function of the osmotic pressure difference over the membrane between bulk DS and bulk FS. Average values calculated based on five separate runs. (**b**) Specific reverse salt flux of the electrospun membrane developed in this work compared to other frequently applied FO membranes CSM [18,31], HTI [32] and FTS [33]. All data measured with demineralized water against 1M NaCl as DS.

During the FO process, water permeates from the FS to the DS due to the osmotic pressure difference between bulk FS and DS. This results in concentration of the FS and dilution of the DS, which in turn decreases the driving force for water permeation. Consequently, in time (i.e., with decreasing osmotic pressure difference; from right to left on *X*-axis) the water flux decreases from ~13 to ~4.6 L/m^2^·h. The consequence of decreased water flux is that the salt depletion in the support layer decreases, a result of which is that the driving force for the reversed salt flux increases (Figure 6). This effect is even fortified in the specific reverse salt flux, where the reverse salt flux is divided by the water flux (Figure 7a).

The specific reverse salt flux is an important characteristic to compare different membranes. Clearly, the electrospun TFC membranes developed in this research have extremely low specific reverse salt flux values, which is even more prominent when compared to other membranes available in the market (Figure 7b). The electrospun membrane shows somewhat lower water flux values compared to the commercial TFC FO membranes. The reverse salt flux, on the other hand, is, with ~0.3 to ~1.5 g/m^2^h, about 10 times lower than that of the commercial membranes. Overall, this results in an extremely low specific reverse salt flux compared to commercial membranes. This very low specific salt flux is a unique property of the electrospun TFC membrane that ensures the concentrated product retains its quality, and that there is no product deterioration and associated loss of commercial value due to salt diffusion from the DS into the concentrated product.

### 3.3. Membrane Performance with Real Industrial Feed Streams

Next, the developed electrospun membranes were used to concentrate apple juice, manure and whey, with osmotic pressures of 20, 15 and 7 bar, respectively (Table 3; [34,35,36]), as feed to investigate their FO performance using industrial feed streams. In FO, the effective water transport from FS to DS ends when the osmotic pressure of the FS is equal to the osmotic pressure of the DS. This equilibrium is achieved from both sides because concentration of the FS goes hand-in-hand with dilution of the DS. Since the concentration of the 3.6 M DS decreases rapidly with time, the DS was refreshed during the measurement series, indicated by the dotted line in the graphs, to maintain a high osmotic drag force. As the obtained water fluxes and concentration factors thus depend on the effective osmotic pressure difference between FS and DS, the effective bulk osmotic pressure differences (i.e., the driving force for water transport) at the different stages of the concentration process for the different feed streams are given in Table 3 as well. These values are used in the discussion below. In this paragraph, first the water flux data in relation to the concentration factors are discussed for each of the different feed streams individually. After that, the FO performance for the three feed streams is compared.

#### 3.3.1. Apple Juice as Feed Stream

**Figure 8:**
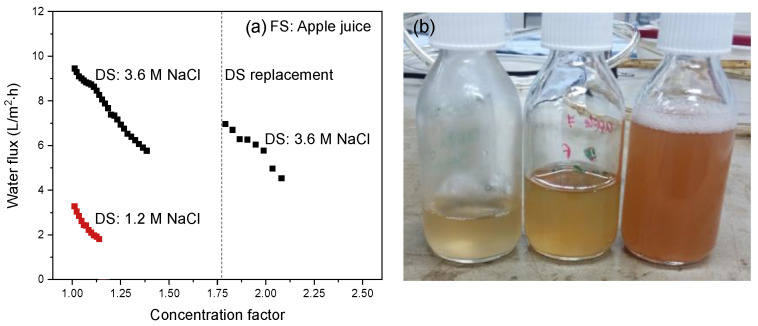
(**a**) Water flux as a function of the concentration factor using apple juice as FS. The intermission in the curve is due to the refreshing of the DS to reset it to 3.6 M NaCl; (**b**) freshly squeezed apple juice (**left bottle**) and the concentrated feed at two stages in the FO-concentration process.

Figure 8 shows the water flux from FS to DS as a function of the associated concentration factor for apple juice concentration using 1.2 or 3.6 M NaCl solution as DS. As the concentration of the 3.6 M DS decreases rapidly in time, the DS was refreshed during the experiment. This is indicated by the dotted line in the curve for the 3.6 M NaCl DS.

The water flux decreases gradually with increasing concentration factor for both the 1.2 and the 3.6 M DS due to the decreasing osmotic pressure difference over the membrane in time as discussed above. In FO with real streams, the water flux is not dictated by the osmotic pressure difference of the bulk solutions but mainly by the concentration present in and at the membrane boundary layer. For the feed side, these effects are mainly ECP and membrane fouling, i.e., accumulation of (apple juice) components in the membrane boundary layer. At the DS side, ICP, depletion of the draw solution due to water permeation, lowers the drag force and thereby the water flux decreases. This effect is most-pronounced at high water fluxes.

When applying a 1.2 M DS, the maximum concentration factor that can potentially be obtained, assuming only water transport, is 1.65. At that point, the osmotic pressure difference between FS and DS is zero. To establish a higher concentration factor a 3.6 M NaCl DS is used (which was refreshed after a concentration factor of 1.8 was obtained, making it theoretically possible to achieve an overall concentration factor of 5.5). In practice, in this research, a concentration factor of 2.1 was obtained after 24 h using a 3.6 M NaCl DS (Table 3) with a still acceptably high water flux of 4.5 L/m^2^·h.

From Figure 8, an initial flux value of ~3.5 L/m^2^·h is obtained for the 1.2 M NaCl DS. Increasing the DS concentration to 3.6 M, the water flux increases by a factor of three. This is somewhat lower than expected since the effective osmotic pressure increases by a factor of 4.7 (Table 3) taking into account the osmotic coefficient and the osmotic pressure of the FS.

This somewhat lower than proportional increase in water flux results from the increased ICP as the result of the higher water flux and accumulation of apple juice components in the boundary layer and on the membrane surface. In conventional FO, membranes with a less permeable support layer ICP often play a dominant role, and this is visible as a far lower than proportional water flux increase with increasing osmotic pressure difference [18]. Replacing the used DS with a fresh 3.6 M NaCl solution partly recovers the water flux, but, as expected, it did not recover to the initial value due to the increased osmotic pressure of the concentrated FS and the presence of membrane fouling. Over the full concentration range, the water flux decreases linearly with the concentration factor, indicating a steady state between, on one side, the crossflow velocity and, on the other side, ECP and membrane fouling.

These results show that apple juice can be concentrated to the desired concentration factor currently obtained with RO (e.g., Álvarez et al. obtained a concentration factor of 2.3 [34]). In addition to the low specific salt flux of the electrospun TFC membrane, it also retains > 98% of the carbon-based components as analyzed with TOC, thus guaranteeing the quality of the final apple juice concentrate. To the best of our knowledge, there are few publications that describe apple juice concentration by FO. Recently, An et al. [20] investigated the concentration of apple juice with a hybrid FO/membrane distillation system. Membrane distillation was used for continuous regeneration of the DS to maintain the driving force. The apple juice was concentrated by a factor 4.25 (76% volume reduction) in 240 h, while in this research we obtained a concentration factor of 2.1 (50% volume reduction) in only 24 h. However, as details on the operational performance of the FO system alone are not available and 2 M potassium sorbate was used as DS, a viable comparison with our work is not possible. What stands out is that the specific salt flux was, with 0.15 g/L, much higher than the specific reverse salt flux obtained in our work for the electrospun TFC membrane (Figure 7), especially as we used NaCl as draw solute, while An et al. [20] used a significantly bigger solute, thus significantly increasing the inherent rejection by the membrane.

#### 3.3.2. Manure as Feed Stream

**Figure 9:**
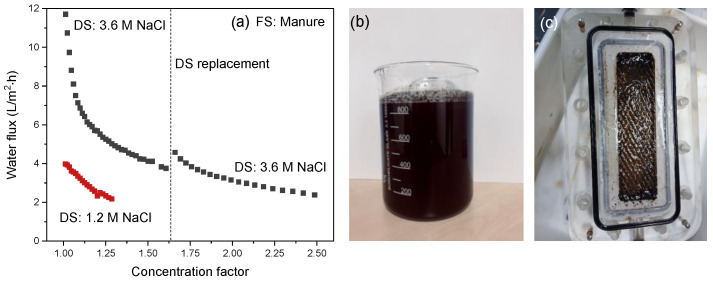
(**a**) Water flux as a function of the concentration factor using manure as FS. The intermission in the curve is due to the refreshing of the DS to reset it to 3.6 M NaCl; (**b**) Initial manure slurry; (**c**) Opened membrane cell after the manure concentration process.

Figure 9 presents the water flux from FS to DS as a function of the corresponding manure concentration factor using a 1.2 or a 3.6 M NaCl solution as DS. As the concentration of the 3.6 M DS decreases rapidly in time, the DS was refreshed during the experiment. This is indicated by the dotted line in the curve for the 3.6 M NaCl DS

Manure concentration with a 1.2 M NaCl DS starts with a water flux of 4 L/m^2^·h that decreases gradually as concentration progresses according to the mechanisms as described in paragraph 4.3. When the initial DS concentration increases from 1.2 M to 3.6 M NaCl, the osmotic pressure difference increases by a factor of 4.3 due to a somewhat lower osmotic pressure of the manure slurry compared to apple juice (Table 3). The initial water flux increases ~3 times, and this again confirms the reduced contribution of ICP for the electrospun support. However, the decrease in water flux with decreasing concentration factor is much steeper compared to apple juice. This is mainly due to the high fouling load of the manure slurry, as the decrease in bulk osmotic pressure at that point is not so strong yet (Table 3). This is confirmed when refreshing the DS: at that point only a slight flux recovery is observed as a consequence of the restored osmotic pressure of the DS. Because at that point the osmotic pressure of the FS has only slightly increased, accumulation of manure components (i.e., coagulants, flocculants, minerals and humic acids) present in the FS boundary layer and as membrane fouling are responsible for an increase in the local osmotic pressure far above the osmotic pressure of the bulk and the increase of the filtration resistance over the membrane.

When compared to concentration factors obtained with RO (i.e., approximately two [37]), our results show that FO is a potentially good alternative for manure concentration, as it can easily obtain concentration factors of 2.5 at reasonable flux values. This is done even without the intensive pretreatments necessary for RO and at fluxes comparable to those in RO processes when operated at 60 bar and high crossflow velocities. Remarkably, the developed electrospun TFC membrane used in these FO experiments also retains 88% of the ammonia present in the manure FS, which is equal to the amount of ammonia that is retained by conventional RO membranes (88%) [37]. In addition, potassium retention of the electrospun TFC membrane was also high (87%). These data show that both ammonia and potassium, both key components in liquid manure fertilizers, are highly retained and remain in the concentrated product, confirming the potential of FO to concentrate highly fouling manure streams for nutrient recovery and use as fertilizer.

A concentration factor of two using manure in FO has been obtained [11]. However, in that case, a very strong decrease in water flux was observed due to very severe fouling of the commercial polyamide membranes. To improve FO performance, research modifying the active membrane layer to decrease its fouling tendency was done, and higher concentration factors (approximately three) in less time (38 h) were obtained, even though the flux values were less than 9 L/m^2^·h [2]. However, as also visible in our work, the high water flux of the electrospun TFC membrane when the 3.6 M NaCl DS is used also increases the drag force on the foulants towards the membrane, thus increasing external concentration polarization in the boundary layer and fouling on the membrane surface, similar to high pressure RO operations, which explains the sudden drop in flux. This shows that, in addition to support improvements, the polyamide selective membrane layer needs to be improved to have maximum benefit of the very porous electrospun support (less ICP) and the high osmotic pressures as driving force over longer concentration times.

#### 3.3.3. Whey as Feed Stream

**Figure 10:**
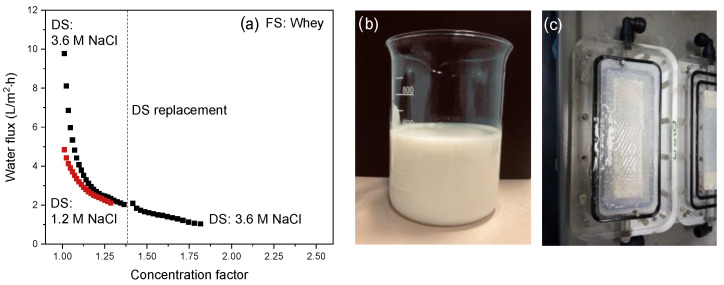
(**a**) Water flux as a function of the concentration factor using whey as FS. The intermission in the curve is due to the refreshing of the DS to reset it to 3.6 M NaCl; (**b**) Initial cheese whey feed; (**c**) Opened membrane cell after the whey concentration process.

Figure 10 shows the water flux from FS to DS in relation to the associated concentration factor to concentrate whey using a 1.2 or a 3.6 M NaCl solution as DS. As previously, the concentration of the 3.6 M DS decreases rapidly in time, and the DS was refreshed during the measurement series. This represents the intermission in the curve for the 3.6 M NaCl DS.

The initial fluxes for a DS of 1.2 and 3.6 M NaCl are ~5 and ~10 L/m^2^·h, respectively. In contrast to what was observed for apple juice and manure, this increase is not proportional to the increase in DS concentration. As the change in osmotic pressure difference over the membrane is only very small, even at these short experiment times, this is due to the strong fouling tendency of whey that virtually instantaneously decreases the water flux both for the experiment with a DS of 1.2 M and the experiment with a DS of 3.6 M (as was also the case for manure with a DS of 3.6 M). A similar strong flux decline when concentrating whey in FO with commercial membranes was observed in our previous study [18]. The flux decline is much stronger for the experiment with a DS of 3.6 M NaCl than for the measurement with a 1.2 M NaCl DS. Opposite to apple juice and manure, the water fluxes for the 1.2 and 3.6 M DSs coincide. This confirms our previous study [18], where we found that for whey, the highest water fluxes were obtained using a DS of 1.2 M. This indicates that fouling deposition is much more severe when higher salt concentrations in the DS are used. The higher initial water flux at higher DS concentrations also increases the drag force of the whey proteins, enhancing their diffusion towards the membrane surface. Simultaneously, the driving force for salt diffusion in the opposite direction (from the DS to the FS) is higher, resulting in higher reverse salt fluxes [18]. This results in a higher amount of salt at the membrane–FS interface and, combined with the accumulation of acid/salt components already present in the native whey feed, induces protein salting-out. Salting-out occurs when the water molecules in the FS are no longer able to surround the charges of the ions and proteins in that solution [38,39]. As a result, hydrophobic interactions between protein molecules become stronger and result in aggregation and, subsequently, denaturation and precipitation of the proteins. Salting out is often initiated close to the membrane surface since the protein concentration is highest there due to the high water-diffusion rates and external concentration polarization (ECP). Simultaneously, the boundary layer at the membrane–solution interface is also the location where the salt concentration is highest due to the high reverse salt flux. These phenomena combined induce protein salting-out and are responsible for the severe membrane fouling and associated strong water flux decline at the initial stages of the experiment.

Irrespective of the DS concentration, similar concentration factors are obtained for both experiments. Replacement of the DS also does not really restore the water flux, confirming that fouling predominantly controls the water-diffusion rate during whey concentration, and that a higher osmotic pressure difference has only little effect (Table 3). The reason of the higher concentration factor for the 3.6 M NaCl DS is solely the longer operational time for concentration (64 h). So in order to reach higher concentration factors, longer operation time or more frequent membrane cleaning [18] is required. Most important to mention here is also that the developed electrospun TFC membranes retain >96% of all carbon-based components (e.g., proteins, amino acids), which means a very low loss of the desired product components to the DS.

In the literature, whey concentration is often studied using hybrid membrane systems where FO is combined with an in-line DS regeneration process to maintain a sufficiently high osmotic pressure gradient over the membrane. In our study, we did not regenerate the DS to obtain more insight into the effect of DS concentration on FO performance. Notwithstanding this, a comparison with the literature will still be given. With a hybrid system including NF/RO for DS regeneration, a concentration factor of 2.5 could be reached [40], which is higher than the values obtained in this work (1.3 for the 1.2 DS and 1.8 for the 3.6 DS). Wang et al. [41] also concentrated whey with FO using a tailor-made TFC membrane with a PA layer applied using interfacial polymerization at the inner side of a poly-ether–sulfone hollow fiber membrane support prepared with non-solvent induced phase separation [42]. Although the ratio feed volume to membrane area was comparable (~300 L/m^2^) in both studies, Wang et al. used an eight-times larger initial DS volume. Furthermore, the corresponding crossflow velocity was, with 55 cm/s, 10 times higher than that applied in our study. This naturally comes at the expense of significantly higher pumping costs and deterioration of shear-sensitive components present in the whey, but, according to Wang et al., it improves the water flux. Even though the active-layer chemistry of both membranes is similar, Wang et al. used DS concentrations of 0.3, 0.5 and 1.0 M only. Because of these low concentrations, severe fouling was not observed, as also indicated in our previous article [18]. The high volume of the DS and the high crossflow velocity may help in this respect, as they prevent a significant drop in osmotic pressure gradient during concentration. In another study, Chen et al. [3] performed an FO pilot test to concentrate whey, giving a concentration factor of 2.5 using a cellulose triacetate FO membrane with an initial water flux of 4.5 L/m^2^·h gradually decreasing to 0.5 L/m^2^·h. This performance is similar to the flux values and concentration factors obtained in our study, but, most importantly, next to the osmotic pressure gradient, they applied a 1 bar hydrostatic pressure difference over the membrane (pressure-assisted forward osmosis; PA-FO), which is known to improve the dewatering performance [43].

**Comparison of Membrane Performance with Real Industrial Feed Streams**

**Figure 11:**
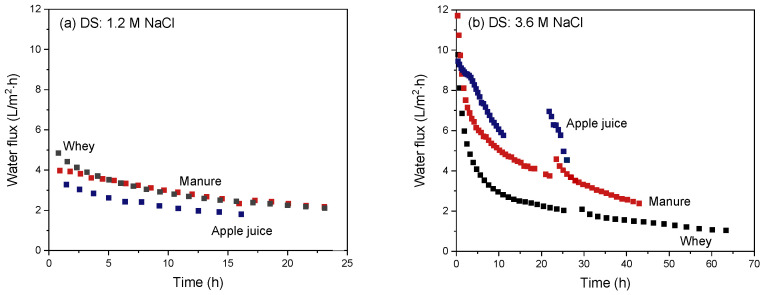
Water flux as a function of the FO operation time using apple juice, manure or whey as FS and using a (**a**) 1.2 M or (**b**) 3.6 M NaCl solution as DS. The intermissions in the curves with 3.6 M NaCl as DS are due to the refreshing of the DS to reset it to 3.6 M NaCl.

Next, the change in flux over time for the three different feeds is compared in Figure 11 for (a) 1.2 M and (b) 3.6 M NaCl as DS.

For all feed streams and DS, the water flux obviously decreases in time due to the decreasing osmotic pressure difference (i.e., the driving force for permeation). Increasing the DS concentration by a factor three from 1.2 M to 3.6 M NaCl initially shows a threefold increase in water flux for all streams. Water flux data over time show that the apple juice at 3.6 M NaCl as DS maintains the highest values and concentration of apple juice at these conditions and is thus faster compared to the other FS (manure and whey). Flux values with manure and whey as FS show a steep initial flux decline despite their higher initial osmotic pressure difference over the membrane.

The original effective osmotic pressures of the FS depend on the FS used: The osmotic pressure of apple juice is ~20 bar [34], that of the used manure stream (conductivity: 27 mS) is ~15 bar [35] and that of whey is ~7 bar [36], ideally generating the highest osmotic pressure difference over the membrane and thus the highest driving force for water transport in the order of least to greatest is apple juice, manure and whey. However, the order in the graphs in Figure 10a and b clearly does not align with that due to the occurrence of membrane boundary layer effects and fouling. As discussed above for the individual streams, apple juice is a relatively low-fouling feed, and ICP and ECP hardly occur as well. In the case of manure, membrane fouling does play a significant role, resulting in a decrease in overall water flux relative to value expected under ideal conditions. In the case of whey concentration, fouling is instantaneous and very severe, in addition also causing ECP, obviously resulting in an even stronger decrease in water flux than ideally expected. This effect is especially very severe at higher DS concentrations due to the previously mentioned salting-out effects [38,39].

**Figure 12:**
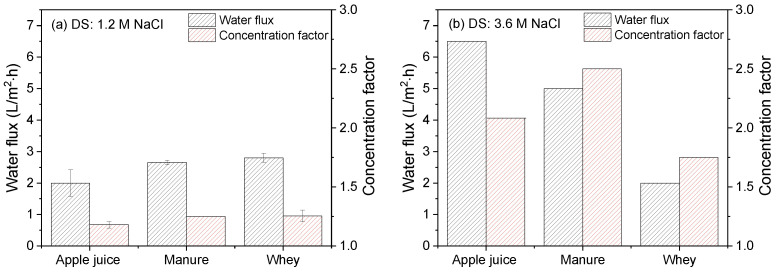
Water flux and concentration factor of industrial apple juice, manure or whey streams as FS and using (**a**) 1.2 M or (**b**) 3.6 M NaCl solution as DS. Average values represent two runs. Concentration time for experiments with 1.2 M NaCl as DS was 24 h, and for experiments with 3.6 M NaCl as DS: apple juice, 24 h; manure, 48 h; whey, 64 h.

Finally, in Figure 12, the water flux relative to the concentration factor for apple juice, manure and whey as FS is compared using (a) 1.2 M NaCl and (b) 3.6 M NaCl as DS in the FO experiments with the developed electrospun TFC membrane.

For all experiments performed with a 1.2 M DS, the average water flux values and concentration factors after FO operation with the developed electrospun TFC membrane are in the same range, irrespective of the FS used. The average water flux when using apple juice indeed increased proportional to the strength of the DS, ending up with a concentrating factor of 2.1. This is a volume reduction of >50% in about 16 h. For the case of apple juice, applying a higher DS concentration is fully utilized, resulting in increased water flux. Correspondingly, for manure, the average water flux was increased by a factor two, reaching a concentration factor of 2.5 in 45 h. In agreement with our previous publication [18], water flux during whey concentration does not benefit from changing the DS from 1.2 to 3.6 M NaCl, showing a decrease in hourly water flux but resulting in a concentration factor of 1.75 after 64 h of operation.

As discussed above, in addition to the osmotic pressure gradient over the membrane as driving force, the use of apple juice gives little fouling feed, while in the case of manure and especially whey as FS membrane, fouling does play a (very) significant role, resulting in a decrease in hourly water flux relative to the expected value under ideal conditions and associated concentration factors. As apple juice has the highest osmotic pressure, thus giving the lowest driving force, the final concentration factor that is obtained is lowest. This also holds for the concentration factors obtained with a 3.6 M DS: due to absence of fouling, hourly water fluxes are high, but, due to the relatively low osmotic pressure difference over the membrane, ultimate concentration factors are lower.

The results presented in this research clearly show the potential for the use of electrospun TFC membranes for FO applications for the dewatering of industrial feed streams. Compared to conventional FO membranes, the very porous, open support prepared with electrospinning decreases ICP significantly. Combined with the ultrathin PA layer applied on top of the support, this can give rise to high water fluxes and high associated concentration factors appropriate for industrial applications. Surprisingly, although manure streams have a very high solids and organic components fraction, manure concentration is especially effective with the developed electrospun TFC membranes, mostly due to an average osmotic pressure difference that gives rise to good flux values while still limiting significant membrane fouling.

## 4. Conclusions

In this work, electrospun TFC membranes with a very porous open support and a dense PA-separating layer are developed, and their performance for the concentration of apple juice, manure and whey in FO is evaluated. The support has a very high porosity of 82.3%, with a mean flow pore size of 2.9 µm. The dense selective PA layer is applied using interfacial polymerization, has a thickness of 0.63 µm and is covalently attached to the support.

When used for FO with UPW as FS and a 1.2 M NaCl solution as DS, the developed electrospun TFC membrane clearly outperforms other conventional FO membranes, and, due to the high flux values and low reverse salt fluxes (0.3–1.5 g/ m^2^·h; about 10 times lower than that of the commercial membranes), its specific reverse salt flux is extremely low, preventing salt diffusion from the DS to the concentrated product stream, thus maintaining product quality.

When concentrating real industrial feeds (apple juice, manure or whey) in FO, the water flux obviously decreases in time due to the decreasing osmotic pressure difference over the membrane. Increasing the DS concentration by a factor three from 1.2 M to 3.6 M NaCl shows a threefold increase in water flux when concentrating apple juice and manure, but less for whey. Apple juice is a relatively low-fouling feed, and also, ICP and ECP hardly occur. During manure concentration, membrane fouling does play a role, but gives a relatively small decrease in overall water flux in time only. Whey concentration results in instantaneous and very severe fouling and ECP, and thus, flux decline, and this effect is especially very severe at high DS concentrations due to protein salting-out effects.

For apple juice, a concentration factor of 2.1 is obtained, i.e., a volume reduction of 50% in about 16 h. For manure, a concentration factor of 2.5 in 45 h is obtained, and for whey a value of 1.75 after 64 h is reached. These values are realistic for industrial applications and in line with what has been reported for other concentration technologies (e.g., RO). This shows the potential of FO to concentrate industrial streams, but also the importance of using membranes with a very open support structure and an ultrathin selective layer, such as the electrospun TFC membrane developed in this work, to maximize FO performance.

## Figures and Tables

**Table 1:** Electrospinning parameters.

Parameter	Value
***Polymer solution***	
Polymer	PSU
Solvent	DMAc
Concentration	22.5 wt.%
Viscosity	2.15 Pa·s
***Electrospinning parameters***	
Relative humidity	25%
Temperature	22.5 °C
Wire-to-wire distance	15 cm
Runner speed	300 mm/s
Runner aperture	0.9 mm
Voltage	40 kV
Wire to substrate distance	25 mm
***Substrate***	
Material	Siliconized paper
Movement speed	30 mm/min

**Table 2:** Characteristics of the custom-made FO membrane.

Parameter	Value
***Porous PSU support layer***	
Thickness	48 ± 12 µm
Fiber diameter	1.18 ± 0.40 µm
Porosity	82.3 ± 5.5%
Mean flow pore size	2.92 ± 0.47 µm
***Dense selective PA top layer***	
Top layer thickness	0.63 µm ± 0.13 µm

**Table 3:** Osmotic pressures of the starting solutions and the effective osmotic pressure during the different stages in time of the concentration process for apple juice, manure and whey concentration.

Osmotic Pressure (Difference)	Apple		Manure		Whey	
Absolute osmotic pressure	p (bar)		p (bar)		p (bar)	
Osmotic pressure FS	20		15		7	
Osmotic pressure DS 3.6 M	193		193		193	
Osmotic pressure difference DS − FS	Dp (bar)	Time (h)				
- Start	172	0				
- At concentration factor 1.4	93	22				
- After DS refreshment	165	22				
- At concentration factor 2.1	321	27				
Osmotic pressure difference DS − FS			Dp (bar)	Time (h)		
- Start			177	0		
- At concentration factor 1.6			82	23		
- After DS refreshment			168	23		
- At concentration factor 2.5			132	43		
Osmotic pressure difference DS − FS					Dp (bar)	Time (h)
- Start					185	0
- At concentration factor 1.4					111	29
- After DS refreshment					180	29
- At concentration factor 1.8					127	63

## Data Availability

Data is contained within the article.
